# Antidiabetic Molecule Efficacy in Patients with Type 2 Diabetes Mellitus—A Real-Life Clinical Practice Study

**DOI:** 10.3390/biomedicines11092455

**Published:** 2023-09-04

**Authors:** Teodor Salmen, Ali Abbas Rizvi, Manfredi Rizzo, Valeria-Anca Pietrosel, Ioana-Cristina Bica, Cosmina Theodora Diaconu, Claudia Gabriela Potcovaru, Bianca-Margareta Salmen, Oana Andreia Coman, Anca Bobircă, Roxana-Adriana Stoica, Anca Pantea Stoian

**Affiliations:** 1 Doctoral School of Carol Davila, University of Medicine and Pharmacy, 020021 Bucharest, Romania; 2 Department of Medicine, University of Central Florida College of Medicine, Orlando, FL 32827, USA; 3 School of Medicine, Department of Health Promotion Sciences Maternal and Infantile Care, Internal Medicine and Medical Specialties (Promise), University of Palermo, 90133 Palermo, Italy; 4 Department of Diabetes, Nutrition and Metabolic Diseases, “Prof. Dr N.C. Paulescu” National Institute of Diabetes, Nutrition and Metabolic Diseases, 030167 Bucharest, Romania; 5 Department of Pharmacology and Pharmacotherapy, Faculty of Medicine, Carol Davila University of Medicine and Pharmacy, 020021 Bucharest, Romania; 6 Internal Medicine and Rheumatology Department, Carol Davila University of Medicine and Pharmacy, 050474 Bucharest, Romania; 7 Department of Diabetes, Nutrition and Metabolic Diseases, Carol Davila University of Medicine and Pharmacy, 050474 Bucharest, Romania

**Keywords:** treatment, real-life, diabetes mellitus, sodium-glucose cotransporter-2 inhibitors, glucagon-like peptide-1 receptor agonist

## Abstract

In this paper, we aim to evaluate the efficacy of antidiabetic cardioprotective molecules such as Sodium-Glucose Cotransporter-2 Inhibitors (SGLT-2i) and Glucagon-like Peptide 1 Receptor Agonists (GLP-1 RAs) when used with other glucose-lowering drugs, lipid-lowering, and blood pressure (BP)-lowering drugs in a real-life setting. A retrospective, observational study on 477 patients admitted consecutively in 2019 to the outpatient clinic of a tertiary care unit for Diabetes Mellitus was conducted. Body mass index (BMI), blood pressure (BP) (both systolic and diastolic), and metabolic parameters, as well as A1c hemoglobin, fasting glycaemia and lipid profile, including total cholesterol (C), HDL-C, LDL-C and triglycerides), were evaluated at baseline and two follow-up visits were scheduled (6 months and 12 months) in order to assess the antidiabetic medication efficacy. Both SGLT-2i and GLP-1 RAs were efficient in terms of weight control reflected by BMI; metabolic control suggested by fasting glycaemia and A1c; and the diastolic component of BP control when comparing the data from the 6 and 12-month visits to the baseline, and when comparing the 12-month visit to the 6-month visit. Moreover, when comparing SGLT-2i and GLP-1 RAs with metformin, there are efficacy data for SGLT-2i at baseline in terms of BMI, fasting glycaemia, and HbA1c. In this retrospective study, both classes of cardioprotective molecules, when used in conjunction with other glucose-lowering, antihypertensive, and lipid-lowering medications, appeared to be efficient in a real-life setting for the management of T2DM.

## 1. Introduction

Modern society is facing an accelerating rate of obesity and type 2 diabetes mellitus (DM) due to changes in diet and lifestyle [1,2]. Longer lifespans and sedentary living are leading to an increase in chronic illnesses that require multiple medications [2]. In this context, polypharmacy is generally referred to as the use of more than five medications per day per patient [3,4]. The high numbers of administered drugs oblige healthcare providers to carefully choose them and, more importantly, to recommend efficient and personalized treatments [5].

Type 2 DM (T2DM) is a complex disease characterized by a hyperglycaemic state with an increased risk of microvascular complications, such as retinopathy, nephropathy, or neuropathy; macrovascular complications such as atherosclerotic disease (peripheral artery disease, ischemic stroke, or coronary artery disease); and cognitive impairment or adverse reactions (AR) from the antidiabetic drugs [6], which all lead to an overwhelming burden. Given the high risk for complications, need for hospitalization, and the all-cause mortality, the current recommendations are to personalize the treatment in order to achieve individualized metabolic targets while addressing the patients’ concomitant comorbidities [6,7].

DM and especially T2DM are characterized by heterogeneity both in pathophysiological and in clinical features, a fact that is emphasized by the recent tendency to cluster patients into subgroups based on disease progression and onset of DM-related complications, including retinopathy, neuropathy, chronic kidney disease, cardiovascular (CV) disease, and NAFLD. Therefore, personalized management of cases, including prevention and treatment methods, should be pursued, but more studies in this direction are required [8].

SGLT-2i and GLP-1 RAs are antidiabetic drugs that are proven to be efficient in achieving glycaemic, metabolic, and weight control, and in reducing the risk of a composite of CV death, nonfatal myocardial infarction (MI), and nonfatal stroke—together referred to as major adverse cardiovascular events (MACE) [9,10].

Randomized control trials (RCTs) represent the gold standard in providing directions for adjusting a patient’s management. Despite their significant usefulness, they require plentiful resources and they offer information on only a select cohort of patients in a more or less controlled setting; therefore, real-life studies are needed in order to provide complementary data to RCTs [11,12].

The aim of this study was to evaluate the efficacy of two classes of glucose-lowering medications, namely SGLT-2i and GLP-1 RAs, for the treatment of T2DM when used in a real-life clinical practice with other glucose, blood pressure (BP), and lipid-lowering medication.

## 2. Materials and Methods

**Figure 1:**
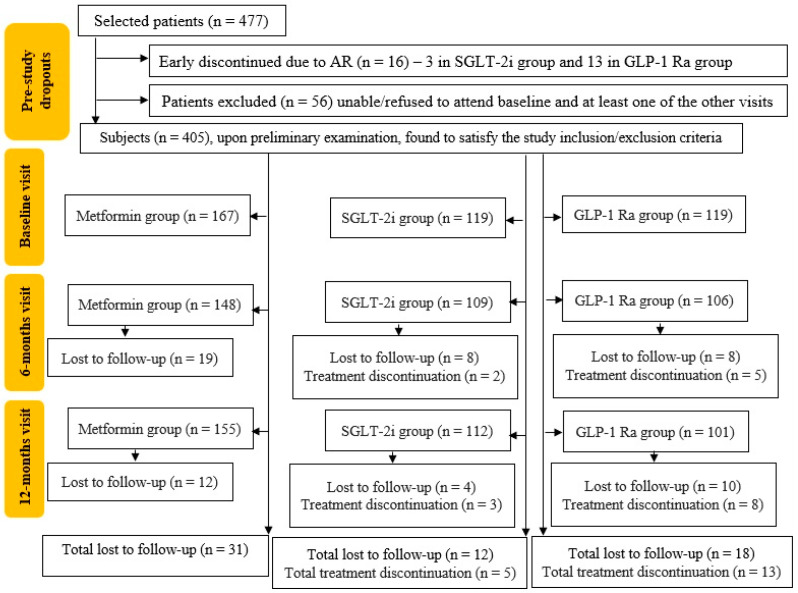
Patient selection and inclusion process. AR—adverse reaction; SGLT-2i—sodium glucose loop transporter 2 inhibitor; GLP-1 RA—glucagon-like peptide 1 receptor agonist.

This retrospective, observational study was conducted in accordance with the Declaration of Helsinki and approved by the Institutional Ethics Committee of N Paulescu National Institute for Diabetes Mellitus, Nutrition and Metabolic Disorders, Bucharest, Romania (protocol number 5591, from 17 November 2022). From the 477 patients that were consecutively admitted in 2019 to the “N. Paulescu” National Institute for Diabetes Mellitus, Nutrition and Metabolic Disorders’ Outpatient Department, 16 patients discontinued their treatment early due to AR, 56 patients refused or were unable to attend baseline visits, and at least one of the control visits and 405 patients met the inclusion criteria. Figure 1 synthetizes the analysis of those patients who was intended to receive treatment, the pre-study drop-outs, those lost for follow-up, and those who discontinued treatment due to AR.

The inclusion criteria are extensively presented in Table 1 and comprise adult patients with at least a 6-month duration of T2DM prior to admission, treated in a standard-of-care regimen for 6 months prior to the baseline visit, and who received at least one of the BP-lowering or lipid-lowering drugs of interest. The included patients had to attend at minimum two of the three visits of interest which were, respectively, a baseline visit (mandatory) (V0M), a plus 6-month visit (V6M) or a plus 12-month visit (V12M), or both a plus V6M and V12M. Furthermore, patients were assigned to one of three groups depending on their non-insulinic treatment for DM which were, respectively, metformin, metformin plus SGLT-2i, and metformin plus GLP-1 Ra. The exclusion criteria are also presented in Table 1 and include non-adult patients with other types of DM.

The drugs of interest from the SGLT-2i and GLP-1 RA classes are the ones that were available and approved by the National Drug Association at the time of the study, beginning with empagliflozin and dapagliflozin for SGLT-2i, and dulaglutide, lixisenatide, and exenatide for GLP-1 RAs.

Patients’ real-life data regarding their demographic parameters (e.g., age, gender, and settlement), clinical examination (BMI, heart rate-HR, systolic, and diastolic BP), comorbidities (e.g., high BP and dyslipidemia, etc.), paraclinical profile (fasting glycaemia, A1c, total-C, HDL-C, LDL-C, and TG), and data about the treatment (antidiabetic, BP-lowering, and lipid-lowering drugs) at V0M, V6M, and V12M were collected from the electronic database of the N. Paulescu National Institute for Diabetes Mellitus, Nutrition and Metabolic Disorders, Bucharest, Romania. Using Excel software 2019th version and SPSS software, 20th version, the data were statistically analyzed using the Kolmogorov–Smirnov test for normality, ANOVA test for baseline characteristics comparison, and student t-test for comparison between visits if the variables had normal distribution, as well as a Wilcoxon test and Kruskal–Wallis tests for non-normal distributions.

## 3. Results

Detailed data regarding the included patients’ participation, demographics, comorbidities, and treatment for the molecules of interest are shown in Table 2. The Romanian standard–of-care treatment regarding the maximum tolerated dose for T2DM for the metformin group is represented by metformin, or metformin plus insulin; in the SGLT-2i group by metformin plus SGLT-2i or metformin plus SGLT-2i plus insulin; in the GLP-1 RA group by metformin plus GLP-1 RAs plus insulin, as shown in Table 2; and, alongside with CV treatment of interest, respectively, beta-blockers (BB), calcium-channel blockers (CCB), angiotensin-converting enzyme inhibitors/angiotensin receptor blockers (ACEI/ARB) or statins and, when needed, diuretics.

The baseline visit (V0M) parameters of interest are the clinical parameters—Body mass index (BMI), heart rate (HR), systolic and diastolic BP, and the metabolic parameters—fasting glycaemia, total-cholesterol (total-C), HDL-cholesterol (HDL-C), LDL-cholesterol (LDL-C) and triglycerides (TG); these are shown in Table 3.

The 6-month visit (V6M) parameters of interest are the clinical parameters—BMI, HR, systolic and diastolic BP, and the metabolic parameters—fasting glycaemia, total-C, HDL-C, LDL-C and TG; these are shown in Table 4.

The 12-month visit (V12M) parameters of interest are the clinical parameters—BMI, HR, systolic and diastolic BP, and the metabolic parameters—fasting glycaemia, total-C, HDL-C, LDL-C, and TG; these are shown in Table 5.

The patients were evaluated both clinically (BMI, systolic and diastolic BP, and HR) and paraclinically (fasting glycaemia, HbA1c, total-C, HDL-C, LDL-C, and TG) at V6M and at V12M as compared to V0M, and at V12M as compared to V6M; the results are synthetized in Table 6. They had at least one statistically significant value with *p* < 0.05, while for systolic BP, HR, total-C, and HDL-C there were no significant differences.

Moreover, a comparison of the SGLT-2i and GLP-1 RA groups with the metformin group for efficacy, looking at BMI, HR, systolic and diastolic BP, HbA1c, fasting glycaemia, total-C, HDL-C, LDL-C, and TG at V0M, at V6M and at V12M are statistically significant only for BMI (3.69 ± 0.73 kg/m^2^, *p* < 0.001), fasting glycaemia (15.27 ± 6.79 mg/dL, *p* = 0.025), and HbA1c (0.72 ± 0.16%, *p* < 0.001) at V0M when comparing SGLT-2i to metformin. Meanwhile, no parameter was efficient when comparing GLP-1 RAs to metformin.

To summarize our results, both SGLT-2i and GLP-1 RA are efficient in terms of weight control, reflected by patients significantly lowering their BMIs after 6 months, with a benefit that was maintained until 12 months. Additionally, metabolic control evaluated by fasting glycaemia and HbA1c improved when comparing the data from V6M and V12M to V0M, and when comparing V12M to V6M; however, only fasting glycemia had a significant decrease after 6 months. Moreover, when comparing SGLT-2i and GLP-1 RAs with metformin, efficacy data were only found for SGLT-2i at V0M for BMI, fasting glycaemia, and HbA1c as compared to metformin.

## 4. Discussion

Our real-world study confirms that, compared to metformin, the antidiabetic non-insulin drugs SGLT-2i and GLP-1 RAs confer extra benefits when administered in standard-of-care treatment and in association with CV drugs used for the treatment of High BP (HBP), such as BB, CCB, ACEI, or ARB, or for the treatment of dyslipidaemia, such as statins. It is important to emphasize that the two classes are reported to have cardioprotective benefits, but the complex mechanisms that lie beyond this property are still being studied.

CVOTs reported that the standard–of-care treatment that included SGLT-2i could provide benefits such as metabolic control by reducing HbA1c [13,14,15], ameliorating hyperglycaemia [14,16], lowering body weight [13,15,16], reducing systolic and diastolic BP [13,14,16], and ameliorating the lipid profile by reducing TG levels [13].

CVOTs that evaluated GLP-1 RAs with the standard–of-care treatment demonstrated that this class has beneficial effects on the reduction of HbA1c [17,18,19], fasting glycaemia [17,18,19,20], body weight [17,18,19], systolic and diastolic BP [17,19], and amelioration of the lipid profile [17,19] by reducing LDL-C, total-C, and TG levels.

Metformin has been used in T2DM as a first-line standard-of-care treatment for several decades [21]. Its benefits were not evaluated by CVOTs [22] because it was widely available with no severe AR, and due to its easy affordability and tolerability [23]. It has beneficial CV effects, as shown in the United Kingdom Prospective Diabetes Study (UKPDS) [23]. It is efficacious in reducing fasting glycaemia [24,25], HbA1c [25], and body weight [25,26], and has modest effects on the lipid profile, especially LDL-C and TG [26]. The following is a narrative comparison of the actions of metformin versus the GLP-1RAs and SGLT-2i agents as reported in the literature and a parallel to our study.

### 4.1. BMI

Metformin, one of the first-line treatment options in T2DM treatment, is reported to reduce weight by inducing satiety and improving insulin sensitivity [25,26,27]. For example, Zyrek et al. [28] reported a reduction in the BMI of patients with T2DM from baseline (27.29 ± 2.1 kg/m^2^) to the 3-month visit (28.27 ± 2.71 kg/m^2^), *p* < 0.0001, which is similar to the findings in our study when comparing BMI at V6M to V0M; V12M to V0M; and V12M to V6M (Table 3, Table 4 and Table 5).

The GLP-1 RAs are a class of antidiabetic drugs used in the treatment of T2DM and have multiple benefits such as increased satiety, reduced appetite and food intake with weight loss, and concomitant gastrointestinal effects such as slowing the gastric emptying rate and small intestinal peristalsis [17,19,20,29]. For this class, Tofé et al. [30] reported a decrease in BMI for patients with T2DM treated with GLP-1 RAs at 6 months (37.05 ± 6.1 kg/m^2^) and at 12 months (37.21 ± 6.8 kg/m^2^) as compared to their initial visit (38.56 ± 6.6 kg/m^2^), *p* < 0.001, results that are similar to the ones from our study when comparing V6M to V0M and V12M to V0M (Table 3, Table 4 and Table 5).

Another aspect is the lack of a significant decrease in BMI between V6M and V12M. This confirms the results of previous studies [31], where the maximum decrease in BMI under GLP-1 RAs is observed after 30 weeks and then is maintained over time.

Another class of antidiabetic drugs with cardioprotective benefits that is used in the treatment of T2DM along with GLP-1 RAs are the SGLT-2i that block SGLT-2-mediated glucose reabsorption in the kidneys, resulting in glycosuria and weight loss [13,15,16,32]. In a study by Sawada et al. [33], there was a decrease in the BMI of patients with T2DM treated with SGLT-2i (without specifying the duration of treatment administration) from 30.3 ± 6.1 kg/m^2^ to 29.2 ± 5.7 kg/m^2^, *p* < 0.001. In our study, SGLT-2i was efficient in terms of reducing BMI at V6M as compared to V0M and at V12M as compared to V0M (Table 3, Table 4 and Table 5).

### 4.2. Blood Pressure

GLP-1 RAs in T2DM are reported to lower BP secondary to weight loss, increase in natriuresis, and provide better regulation of the renin–angiotensin–aldosterone system [17,19,29,34,35]. In a study by Hu et al. [35] a reduction in diastolic BP of −0.898 mmHg, *p* < 0.001, was reported in patients with T2DM treated with GLP-1 RAs, consistent with our study results, where we found a reduction at V6M as compared to V0M and V12M as compared to V0M (Table 3, Table 4 and Table 5).

BP reduction in the SGLT-2i class of patients with T2DM can be explained by decreased sodium reabsorption in the proximal renal tubule, increase in diuresis with a reduction in the plasma volume, improved arterial stiffness, and by the indirect effect of weight reduction [36,37]. The data reported by Sawada et al. [33] showed a decrease in diastolic BP in patients with T2DM treated with SGLT-2i from 74 ± 12 mmHg before initiation to 71 ± 12 mmHg afterwards, *p* = 0.332, but they did not state the duration of the follow-up.

### 4.3. Fasting Glycaemia

Metformin ameliorates fasting glycaemia in patients with T2DM by decreasing the hepatic glucose production and the production of reactive oxygen species, resulting in an improvement in cerebral memory and cognitive performance, along with glycaemic control [24,25,38]. In a study by Rosenstock et al. [39], there was a reported reduction in plasma glycaemic levels in patients with T2DM treated with metformin (191 ± 49 mg/dL) as compared to levels during the 26-week visit (mean reduction −35 ± 3 mg/dL). Interestingly, this is similar to our results from V6M as compared to V0M; V12M as compared to V0M; and at V12M as compared to V6M (Table 3, Table 4 and Table 5).

SGLT-2i reduces proximal glucose reabsorption in the kidney, leading to a decrease in blood glucose levels when used in patients with T2DM [40]. Singh et al. [41] reported that SGLT-2i used in the treatment of T2DM has durable efficiency in reducing glycaemic levels, which is consistent with our findings at V6M as compared to V0M; at V12M as compared to V0M; and at V12M as compared to V6M (Table 3, Table 4 and Table 5).

GLP-1 RAs ameliorate the glycaemic profile in patients with T2DM by increasing the secretion of insulin and synthesis of pancreatic islet cells, in parallel with a decrease in glucagon secretion and β-cell apoptosis [17,18,19,20,34]. In a study conducted by Tofé et al. [30] in patients with T2DM, treatment with GLP-1 RAs was efficient in reducing fasting glycaemia at 6 months (145 ± 51 mg/dL) and at 12 months (153 ± 53 mg/dL) as compared to the initial visit (177 ± 59 mg/dL), *p* < 0.0001. In our study, the differences in terms of glycaemic control were encountered at V6M as compared to V0M; at V12M as compared to V0M; and at V12M as compared to V6M (Table 3, Table 4 and Table 5).

### 4.4. HbA1c

Metformin reduces HbA1c in patients with T2DM [25]. Rosenstock et al. [39] reported that in patients with T2DM with initial HbA1C ≥ 8%, metformin therapy led to a reduction in HbA1c from 8.70 ± 0.033% to 7.57 ± 0.08% at 3 months, *p* < 0.0001, which is consistent with our results showing HbA1c at V6M as compared to V0M (Table 3, Table 4 and Table 5).

GLP-1 RAs are credited with glucose-lowering effects and with an approximately 1% reduction in HbA1c when used in patients with T2DM [17,18,19,34]. Tofé et al. [30] reported that GLP-1 RA therapy reduced HbA1c at 6 months (7.24 ± 1.45%) and at 12 months (7.29 ± 1.51%) as compared to the initial visit (8.18 ± 1.53%), *p* < 0.0001, in subjects with T2DM—results that are similar to our study when comparing V6M to V0M (Table 3, Table 4 and Table 5).

SGLT-2is are reported to reduce HbA1c in patients with T2DM with values that range from 0.5% to 1% [42], but the reduction can be larger as, for example, in a meta-analysis by Masson W et al., where they reported a reduction in HbA1c of −0.94% 95% CI (−1.69, −0.18), *p* = 0.002 [43], results that are similar to our study when comparing V6M with V0M and V12M with V6M.

It is important to emphasize that a possible explanation for the lower improvement in HbA1c between V12M and V6M as compared to V6M as compared to baseline could be secondary to the lower HbA1c from the baseline.

### 4.5. SGLT-2i, GLP-1 RAs and Metformin Comparison

Optimal management of T2DM frequently requires combination therapy with several glucose-lowering drugs. Since metformin has a long track record, it has been generally accepted as a safe and effective first-line therapy by international consensus guidelines and recommendations. Our study showed the efficacy of both SGLT-2i and GLP-1 RAs only for fasting glycaemia when compared to metformin in the treatment of T2DM as standard-of-care in patients with T2DM. The data from the literature indicate that they are also efficient for metabolic control (HbA1c, fasting glycaemia), body weight, BP lowering, and improving the lipid profile. A systematic review and meta-analysis by Milder et al. [44] compared the combination of SGLT-2i and metformin with metformin alone in the treatment of T2DM. Differences in efficacy were observed for HbA1c, with a 95% confidence interval (95% CI) of −0.55% (−0.67, −0.43), body weight with a 95% CI of –2 kg (−2.34, −1.66), and systolic and diastolic BP reduction. In a meta-analysis that also compared the differences in efficacy between SGLT-2i plus metformin with metformin alone in the treatment of T2DM, Jinfang et al. [45] reported a reduction in HbA1c with 95% CI of −0.50% (−0.62, −0.38), weight gain with a 95% CI of −1.72kg (−2.05, −1.39), systolic BP with a 95% CI of −4.44 mm Hg (−5.45, −3.43), and diastolic BP with a 95% CI of −1.74 mm Hg (−2.40, −1.07), as well as fasting plasma glucose levels of −20.16 mg/dL with a 95% CI of (−24.84, −15.66). The DISCOVER study by Khunti et al. [46] evaluated the association of metformin with sulphonylurea (SU), dipeptidyl peptidase-4 (DPP-4) inhibitors, SGLT-2i, or GLP-1 RAs and reported a significantly reduction in HbA1c and body weight for the last three groups as compared to the first one.

In a study by Hutmacher et al. [47], GLP-1 RAs plus basal insulin reduced HbA1c by −0.7% (95% CI −1.2, −0.2) when compared with basal insulin and placebo; the effects of long-acting GLP-1 RAs (−1.0%, 95% CI −1.2, −0.8) and of short-acting GLP-1 RAs (−0.5%, 95% CI −1.2, −0.8) were similar. However, it is generally believed that long-acting GLP-1 RAs are more efficient in terms of reduction in HbA1c, fasting plasma glycaemia, and body weight when compared to short-acting GLP-1 RAs.

A systematic review and meta-analysis by Patoulias et al. [48] reported that GLP-1 RAs offered better HbA1c reduction with −0.38% (95% CI −0.55, −0.22) as compared to SGLT-2i, but with similar improvements in body weight −0.29 kg (95% CI −1.24, 0.66) and fasting plasma glycaemia in T2DM. GLP-1 RAs are not superior to SGLT-2i for systolic BP 0.98 (95% CI −1, 2.97) and for diastolic BP 1.01 (95% CI −0.25, 2.27).

### 4.6. Future Perspectives in Efficacy of Cardioprotective Molecules

Taking into consideration the evolution of modern medicine towards precision treatment, including in the case of DM and especially in T2DM, we should not forget that the efficacy of any treatment should be reported to the type of the patient [8]. Future studies should analyze if the drugs provide different grades of efficacy due to their variable curves of efficacy or due to different cut-offs until which the patients respond, or due to the different pathophysiology of DM, different DM evolution patterns, complication onsets, or comorbidities of the patients [49,50].

Moreover, in addition to the present real-life clinical practice results in efficacy for cardioprotective antidiabetic drugs, we reported in a previous paper [51] data about their safety as part of the same research project. Clinical inertia may be the main cause for low rates of success in achieving metabolic control. However, in this project, we did not select the included patients, but enrolled them consecutively, and clinicians tend to indirectly select the patients that receive these drugs based on their medical judgement, despite the guideline recommendations and the need to move the paradigm from HbA1c control to a comprehensive metabolic control that targets weight control, lipid profile, or BP control [52], which allow for a reduction in CV risk.

Thus, there is hope for patients with T2DM because recently, in randomized multicenter, clinical studies, it was reported that the reduction in MACEs and mortality is incrementally related to the number of risk factors that reached the recommended targets [53,54].

The strengths of our study rely on gaining real-life clinical practice data for molecules that once more proves their benefits in body weight and BP control, and in helping to achieve a lipid profile and metabolic profile closer to the recommended ones as compared to the CVOT data, where the patients are more carefully selected, monitored, and treated. On the other hand, the limitations of our study include that, despite a robust association, its observational design precludes establishing a definite causal relationship, along with a relatively small number of subjects. The short period of follow-up of a maximum of three visits in a one-year duration can also explain why the only discontinuation cause was AR, while the real-life conditions brought up dietary and physical activity variation between patients, as well as different grades of adherence, which are difficult to quantify, but may influence the results; the baseline differences between treatment groups and patient selection by the diabetologists may include preferential selection of patients who are more adherent to the medical recommendations, due to the high financial burden that the treatment represents for the national health system. However, our results are promising and provide the basis for larger, randomized studies in this therapeutic area and in real-life settings, which can be performed on a longer duration to evaluate the maintenance of the effect in time or to figure out several explanations for poor efficacy, including the segregation by clusters of T2DM patients.

## 5. Conclusions

The present real-life study presents two classes of noninsulin antidiabetic agents, namely GLP-1 RAs and the SGLT-2i, which appear to be efficacious in the reduction in body weight reflected by BMI at 6 and 12 months as compared to baseline, along with metabolic control reflected by reducing fasting glycaemia at 6 and 12 months as compared to baseline and at 12 months as compared to the 6-month visit, and by reducing HbA1c at 6 months as compared to baseline visit when used in a real-life clinical practice setting for patients with T2DM, even in combination with therapeutic agents for treating HBP (BB, CCB, ACEI, or ARB) or for the treatment of dyslipidaemia with statins. Therefore, this study adds to the body of literature, and is close to real-world, clinical, and translational care, showing that the resultant multifactorial reduction in CV risk may prove to be highly beneficial in reducing morbidity and mortality in patients with T2DM.

## Figures and Tables

**Table 1:** Inclusion and exclusion criteria.

Inclusion Criteria	Exclusion Criteria
Adults > 18 years old	Younger than 18 years old
Duration of T2DM > 6 months	Type 1 DM or secondary DM
Standard-of-care treatment for T2DM with maximum tolerated doses > 6 months prior to inclusion	Severe/acute heart failure, renal insufficiency or hepatic insufficiency
At least two visits from V0M, V6M and V12M	
Treatment with BB and/or CCB and/or ARB/ACEI and/or statin	

DM—diabetes mellitus; T2DM—type 2 diabetes mellitus; V0M—baseline visit; V6M—6 months visit; V12M—12 months visit; BB—beta-blockers; CCB—calcium-channel blockers; ARB—angiotensin receptor blockers; ACEI—angiotensin converting enzyme inhibitors.

**Table 2:** Data about the patient’s participation at visits, demographic and standard-of-care treatment for T2DM and cardiovascular (CV) treatment of interest.

	Metformin	SGLT-2i	GLP-1Ras	
No of patients (% of total)	167 (41.2%)	119 (29.4%)	119 (29.4%)	
No of patients at V6M (% of group)	148 (88.62%)	109 (91.59%)	106 (89.07%)	
No of patients at V12M (% of group)	155 (92.81%)	112 (94.11%)	101 (84.87%)	
Insulin treatment (%)	16 (9.58%)	15 (12.6%)	61 (51.26%)	*p* < 0.001, η^2^ = 0.24
Female (%)	65 (38.9%)	34 (71.4%)	57 (47.9%)	*p* = 0.009, η^2^ = 0.023
Mean age (years) [mean ± SD]	57 ± 10	56 ± 10	59 ± 9	*p* = 0.185, η^2^ = 0.008
Urban settlement (%)	113 (67.66%)	98 (82.35%)	88 (73.95%)	*p* < 0.001, η^2^ < 0.001
Active smoker (%)	29 (17.36%)	15 (12.6%)	27 (22.68%)	*p* = 0.281, η^2^ = 0.006
Chronic kidney disease (%)	14 (8.38%)	15 (12.6%)	20 (16.80%)	*p* = 0.097, η^2^ = 0.012
Heart failure (%)	10 (5.98%)	14 (11.76%)	8 (6.72%)	*p* = 0.174, η^2^ = 0.009
BB (%)	94 (56.28%)	78 (65.54%)	74 (62.18%)	*p* = 0.268, η^2^ = 0.007
CCB (%)	43 (25.74%)	28 (23.52%)	28 (23.52%)	*p* = 0.818, η^2^ = 0.01
ACEI/ARB (%)	104 (62.27%)	84 (70.58%)	94 (78.98%)	*p* = 0.01, η^2^ = 0.023
Statin (%)	135 (80.83%)	104 (87.39%)	106 (89%)	*p* = 0.112, η^2^ = 0.011
Diuretics (%)	73 (43.71%)	36 (30.25%)	43 (36.13%)	*p* = 0.181, η^2^ = 0.008

SGLT-2i—sodium glucose loop transporter 2 inhibitor; GLP-1 Ras—glucagon-like peptide 1 receptor agonist; V6M—6-month visit; V12M—12-month visits; BB—beta-blockers; CCB—calcium channel blockers; ACEI—angiotensin converting enzyme inhibitors; ARB—angiotensin receptor blockers; SD—standard deviation.

**Table 3:** The V0M parameters of interest for clinical (BMI, HR, systolic and diastolic BP) and metabolic parameters (fasting glycaemia, total-C, HDL-C, LDL-C and TG).

	Metformin (n = 167)		SGLT-2i (n = 119)		GLP-1Ras (n = 119)		
BMI (kg/m^2^) [mean ± SD]	31.8 ± 5.8	*p* = 0.672	35.5 ± 6.5	*p* = 0.209	32.1 ± 6.1	*p* = 0.022	*p* < 0.001, η^2^ = 0.067
Systolic BP (mmHg) [mean ± SD]	133.4 ± 12.8	*p* = 0.004	131.7 ± 13.4	*p* = 0.009	131.4 ± 1	*p* = 0.022	*p* = 0.377, η^2^ = 0.05
Diastolic BP (mmHg) [mean ± SD]	80.4 ± 8.7	*p* < 0.001	79.7 ± 12.7	*p* < 0.001	79.3 ± 8.8	*p* < 0.001	*p* = 0.63, η^2^ = 0.002
HR (beat per minute) [mean ± SD]	78 ± 11	*p* < 0.001	77 ± 12	*p* = 0.009	73 ± 8	*p* = 0.23	*p* < 0.001, η^2^ = 0.041
Fasting glycaemia (mg/dL) [mean ± SD]	155.4 ± 48.4	*p* = 0.026	170.6 ± 66.1	*p* = 0.006	155.7 ± 49.5	*p* = 0.003	*p* = 0.041, η^2^ = 0.016
HbA1c (%) [mean ± SD]	7.4 ± 1.2	*p* = 0.006	8.1 ± 1.5	*p* = 0.068	7.4 ± 1.4	*p* = 0.044	*p* < 0.001, η^2^ = 0.05
Total-C (mg/dL) *	172 (52)	*p* = 0.236	161 (61)	*p* = 0.595	168 (60)	*p* = 0.654	*p* = 0.215, η^2^ = 0.008
HDL-C (mg/dL) [mean ± SD]	46 ± 12	*p* = 0.008	43 ± 16	*p* < 0.001	44 ± 13	*p* = 0.11	*p* = 0.182, η^2^ = 0.008
LDL-C (mg/dL) *	92 (41)	*p* = 0.232	98 (47)	*p* = 0.168	91 (53)	*p* = 0.403	*p* = 0.816, η^2^ = 0.001
TG (mg/dL) *	170 (103)	*p* = 0.004	172 (115)	*p* = 0.002	170 (82)	*p* < 0.001	*p* = 0.419, η^2^ = 0.004

SGLT-2i—sodium glucose loop transporter 2 inhibitor; GLP-1 RA—glucagon like peptide 1 receptor agonist; total-cholesterol—total-C; HDL-cholesterol—HDL-C; LDL-cholesterol—LDL-C; TG—triglycerides; SD—standard deviation. *—where the baseline distribution was not normal, we reported the data as median and interquartile range.

**Table 4:** The V6M parameters of interest for clinical (BMI, HR, systolic and diastolic BP) and metabolic parameters (fasting glycaemia, total-C, HDL-C, LDL-C, TG).

	Metformin (n = 148)		SGLT-2i (n = 109)		GLP-1Ras (n = 106)	
BMI (kg/m^2^) [mean ± SD]	31.3 ± 5.9	*p* = 0.756	31.6 ± 5.5	*p* = 0.96	31.5 ± 5.8	*p* = 0.022
Systolic BP (mmHg) [mean ± SD]	133.1 ± 12.8	*p* = 0.004	133.1 ± 12.8	*p* = 0.017	130.7 ± 16.1	*p* = 0.08
Diastolic BP (mmHg) [mean ± SD]	80.8 ± 9.9	*p* < 0.001	81.6 ± 10.1	*p* < 0.001	76.9 ± 9.7	*p* < 0.001
HR (beat per minute) [mean ± SD]	77 ± 9	*p* = 0.009	76 ± 9	*p* = 0.015	73 ± 8	*p* = 0.782
Fasting glycaemia (mg/dL) [mean ± SD]	136.8 ± 36.1	*p* = 0.357	133.9 ± 35.3	*p* = 0.282	135.4 ± 35.3	*p* = 0.099
HbA1c (%) [mean ± SD]	7.1 ± 1.2	*p* = 0.002	7 ± 1.2	*p* = 0.027	7.1 ± 1.3	*p* = 0.02
Total-C (mg/dL) *	170 (60)	*p* = 0.32	167 (61)	*p* = 0.23	164 (55)	*p* = 0.345
HDL-C (mg/dL) [mean ± SD]	46 ± 11	*p* = 0.029	47 ± 12	*p* = 0.117	45 ± 11	*p* = 0.855
LDL-C (mg/dL) *	92 (52)	*p* = 0.425	88 (50)	*p* = 0.266	90 (50)	*p* = 0.081
TG (mg/dL) *	156 (120)	*p* = 0.132	142 (123)	*p* = 0.171	154 (91)	*p* = 0.026

SGLT-2i—sodium glucose loop transporter 2 inhibitor; GLP-1 RA—glucagon like peptide 1 receptor agonist; total-cholesterol—total-C; HDL-cholesterol—HDL-C; LDL-cholesterol—LDL-C; TG—triglycerides; SD—standard deviation. *—where the baseline distribution was not normal, we reported the data as median and interquartile range.

**Table 5:** The V12M parameters of interest for clinical (BMI, HR, systolic and diastolic BP) and metabolic parameters (fasting glycaemia, total-C, HDL-C, LDL-C, TG).

	Metformin (n = 155)		SGLT-2i (n = 112)		GLP-1RAs (n = 101)	
BMI (kg/m^2^) [mean ± SD]	31.0 ± 5.8	*p* = 0.582	31.2 ± 5.4	*p* = 0.78	31.3 ± 5.7	*p* = 0.05
Systolic BP (mmHg) [mean ± SD]	133.2 ± 13.0	*p* = 0.005	132 ± 12.7	*p* = 0.011	130 ± 13.6	*p* = 0.029
Diastolic BP (mmHg) [mean ± SD]	79.9 ± 10.7	*p* < 0.001	80.3 ± 11.4	*p* < 0.001	76.5 ± 10.3	*p* < 0.001
HR (beat per minute) [mean ± SD]	77 ± 9	*p* = 0.063	77 ± 9	*p* = 0.03	73 ± 9	*p* = 0.327
Fasting glycaemia (mg/dL) [mean ± SD]	142.9 ± 39.9	*p* = 0.157	139.2 ± 36.8	*p* = 0.262	146 ± 50.7	*p* = 0.007
HbA1c (%) [mean ± SD]	7.1 ± 1.1	*p* = 0.01	7 ± 1	*p* = 0.073	7.1 ± 1	*p* = 0.023
Total-C (mg/dL) *	173 (57)	*p* = 0.035	165 (58)	*p* = 0.053	166 (62)	*p* = 0.508
HDL-C (mg/dL) [mean ± SD]	47 ± 12	*p* = 0.019	48 ± 12	*p* = 0.096	45 ± 13	*p* = 0.212
LDL-C (mg/dL) *	92 (43)	*p* = 0.064	87 (39)	*p* = 0.091	91 (50)	*p* = 0.504
TG (mg/dL) *	162 (103)	*p* = 0.017	140 (100)	*p* = 0.095	152 (85)	*p* = 0.017

SGLT-2i—sodium glucose loop transporter 2 inhibitor; GLP-1 RA—glucagon like peptide 1 receptor agonist; total-cholesterol—total-C; HDL-cholesterol—HDL-C; LDL-cholesterol—LDL-C; TG—triglycerides; SD—standard deviation. *—where the baseline distribution was not normal, we reported the data as median and interquartile range.

**Table 6:** Clinical (BMI, diastolic BP) and paraclinical (fasting glycaemia, HbA1c, HDL-cholesterol and tryglicerides) at V6M and at V12M as compared to V0M, and at V12M as compared to V6M.

	Metformin		SGLT-2i		GLP-1 RAs	
	Mean Difference		Mean Difference		Mean Difference	
V6M compared to V0M						
BMI (kg/m^2^)	0.5 ± 0.09	*p* < 0.001	3.9 ± 0.78	*p* < 0.001	0.6 ± 0.1	*p* < 0.001
Diastolic BP (mmHg)	0.4 ± 0.8	*p* = 0.380	1.9 ± 1.3	*p* = 0.151	2.4 ± 0.8	*p* = 0.013
Fasting glycaemia (mg/dL)	18.6 ± 3.8	*p* < 0.001	36.7 ± 7.4	*p* < 0.001	20.3 ± 4.6	*p* < 0.001
HbA1c (%)	0.3 ± 0.1	*p* = 0.018	1.1 ± 0.2	*p* < 0.001	0.3 ± 0.1	*p* = 0.01
HDL-cholesterol (mg/dL)	0 ± 0.6	*p* = 0.765	4 ± 2	*p* < 0.001	0 ± 0.8	*p* = 0.895
Triglycerides (mg/dL)	4 ± 8.3	*p* = 0.38	30 ± 12.5	*p* = 0.023	2 ± 12.2	*p* = 0.258
V12M compared to V0M						
BMI (kg/m^2^)	0.3 ± 0.1	*p* < 0.001	4.3 ± 0.75	*p* < 0.001	0.8 ± 0.1	*p* < 0.001
Diastolic BP (mmHg)	0.9 ± 0.9	*p* = 0.728	0.6 ± 1.3	*p* = 0.314	2.8 ± 1.1	*p* = 0.008
Fasting glycaemia (mg/dL)	6.1 ± 4	*p* = 0.018	31.4 ± 7.2	*p* = 0.001	9.7 ± 5.2	*p* = 0.05
HbA1c (%)	0 ± 0.08	*p* = 0.195	1.1 ± 0.1	*p* < 0.001	0.3 ± 0.1	*p* = 0.075
HDL-cholesterol (mg/dL)	1 ± 0.7	*p* = 0.056	5 ± 2	*p* < 0.001	1 ± 0.6	*p* = 0.283
Triglycerides (mg/dL)	8 ± 8.1	*p* = 0.906	32 ± 7.2	*p* = 0.019	28 ± 12.9	*p* = 0.099
V12M compared to V6M						
BMI (kg/m^2^)	0.8 ± 0.08	*p* = 0.04	4.3 ± 0.09	*p* = 0.086	0.2 ± 0.09	*p* = 0.083
Diastolic BP (mmHg)	0.5 ± 1	*p* = 0.67	1.3 ± 1.3	*p* = 0.759	0.4 ± 1.1	*p* = 0.322
Fasting glycaemia (mg/dL)	12.5 ± 3	*p* = 0.024	31.4 ± 3.3	*p* = 0.045	10.6 ± 3.5	*p* = 0.025
HbA1c (%)	0.3 ± 0.07	*p* = 0.426	0 ± 0.9	*p* = 0.952	0 ± 0	*p* = 0.24
HDL-cholesterol (mg/dL)	1 ± 0.6	*p* = 0.342	1 ± 0.6	*p* = 0.536	1 ± 0.8	*p* = 0.442
Triglycerides (mg/dL)	6 ± 0.1	*p* = 0.51	2 ± 12.5	*p* = 0.974	16 ± 8.1	*p* = 0.798

SGLT-2i—sodium glucose loop transporter 2 inhibitor; GLP-1 RAs—glucagon like peptide 1 receptor agonist; V0M—baseline visit; V6M—6-month visit; V12M—12-month visit; BMI—body mass index; BP- blood pressure; HR—heart rate; Chol -cholesterol; TG—triglycerides.

## Data Availability

Data available on request from the corresponding authors.
